# Rotated domains in selective area epitaxy grown Zn_3_P_2_: formation mechanism and functionality†

**DOI:** 10.1039/d1nr06190a

**Published:** 2021-10-30

**Authors:** Maria Chiara Spadaro, Simon Escobar Steinvall, Nelson Y. Dzade, Sara Martí-Sánchez, Pol Torres-Vila, Elias Z. Stutz, Mahdi Zamani, Rajrupa Paul, Jean-Baptiste Leran, Anna Fontcuberta i Morral, Jordi Arbiol

**Affiliations:** Catalan Institute of Nanoscience and Nanotechnology (ICN2), CSIC and BIST Campus UAB Bellaterra Barcelona Catalonia 08193 Spain arbiol@icrea.cat; Laboratory of Semiconductor Materials, Institute of Materials, Faculty of Engineering, Ecole Polytechnique Fédérale de Lausanne 1015 Lausanne Switzerland anna.fontcuberta-morral@epfl.ch; School of Chemistry, Cardiff University Main Building Park Place CF10 3AT Cardiff UK; Department of Energy and Mineral Engineering, Pennsylvania State University University Park PA 16802 USA; Institute of Physics, Faculty of Basic Sciences, Ecole Polytechnique Fédérale de Lausanne 1015 Lausanne Switzerland; ICREA Pg. Lluís Companys 23 08010 Barcelona Catalonia Spain

## Abstract

Zinc phosphide (Zn_3_P_2_) is an ideal absorber candidate for solar cells thanks to its direct bandgap, earth-abundance, and optoelectronic characteristics, albeit it has been insufficiently investigated due to limitations in the fabrication of high-quality material. It is possible to overcome these factors by obtaining the material as nanostructures, *e.g. via* the selective area epitaxy approach, enabling additional strain relaxation mechanisms and minimizing the interface area. We demonstrate that Zn_3_P_2_ nanowires grow mostly defect-free when growth is oriented along the [100] and [110] of the crystal, which is obtained in nanoscale openings along the [110] and [010] on InP(100). We detect the presence of two stable rotated crystal domains that coexist in the structure. They are due to a change in the growth facet, which originates either from the island formation and merging in the initial stages of growth or lateral overgrowth. These domains have been visualized through 3D atomic models and confirmed with image simulations of the atomic scale electron micrographs. Density functional theory simulations describe the rotated domains’ formation mechanism and demonstrate their lattice-matched epitaxial relation. In addition, the energies of the shallow states predicted closely agree with transition energies observed by experimental studies and offer a potential origin for these defect transitions. Our study represents an important step forward in the understanding of Zn_3_P_2_ and thus for the realisation of solar cells to respond to the present call for sustainable photovoltaic technology.

## Abstract 2

Growth process for Zn_3_P_2_ nanowires grown on InP at different angles by SAE and formation of rotated domains at (100) and (101) facets.

## Graphical abstract

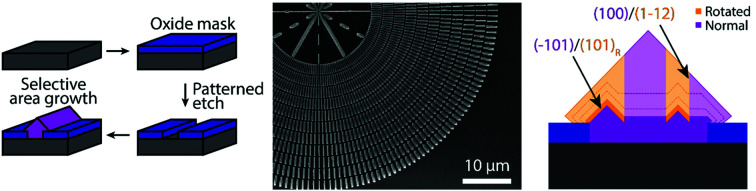

## Introduction

Nanoscale approaches are increasingly used to help circumvent challenges experienced by certain materials if they were to be used in their bulk form. One such material is zinc phosphide (Zn_3_P_2_), a potential photovoltaic absorber (*E*_g_ ∼1.50 eV) or IR-optoelectronics material which has experienced limitations due to its lattice parameter and coefficient of thermal expansion (CTE) not matching commonly available substrates, thus complicating high-quality epitaxial growth.^1–6^ What makes Zn_3_P_2_ attractive is the earth-abundance of the constituent elements,^7^ its direct bandgap close to the optimum for photovoltaic applications,^8^ high optical absorption in the visible range (10^4^–10^5^ cm^−1^),^9,10^ and long minority carrier diffusion length,^8^ which combined hold promise to make this a high-impact and sustainable photovoltaic material.

Zn_3_P_2_ has a tetragonal crystal structure belonging to the *P*4_2_/*nmc* space group (*a* = 8.089 Å, *c* = 11.39 Å), and a CTE of 1.4 × 10^−5^ K^−1^, which is an order of magnitude larger than commercial semiconductors (*e.g.* Si 2.96 × 10^−6^ K^−1^) and more closely matched to that of steel (1.1–1.3 × 10^−5^ K^−1^ depending on exact composition).^2,11–13^ These factors complicate epitaxial growth without a high number of misfit dislocations on commercially available substrates. However, the large size of the unit cell is to account for the ordering of the ordered empty sites in what would otherwise be a cubic zinc sublattice.^14–17^ Therefore, as 
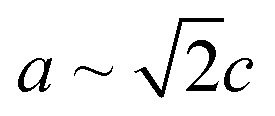
, it is possible to create a pseudo-cubic reconstruction of the unit cell by disregarding the ordering of these sites with a lattice parameter of 5.72 Å, which has been shown to be relevant in the interface ordering during epitaxial growth.^16,18^ It has thus been shown to grow epitaxially on GaAs and InP substrates.^1,16,18,19^ Growth on GaAs resulted in an interface GaP layer which was detrimental to device performance, making InP the ideal system for epitaxial growth studies of Zn_3_P_2_.^20^

Additional methods to facilitate growth of Zn_3_P_2_ include molecular beam epitaxy (MBE) and its growth in the form of nanostructures.^1,3,6,16,17^ MBE growth of Zn_3_P_2_ utilises relatively low growth temperatures as compared to other commonly used techniques.^21^ The lower growth temperatures help mitigate the potential post-growth strain build up due to mismatching CTE, which could otherwise result in defect formation of the grown material. Nanostructures, on the other hand, have been shown to allow for additional elastic strain relaxation mechanisms due to their reduced dimensions, as well as supressing misfit dislocation formation due to their limited interface area (efficiency being a function of lattice mismatch and interface area).^22–28^ One of the main approaches for the epitaxial growth of nanostructures is selective area epitaxy (SAE), which utilises a mask to limit the growth to certain (nanoscale) openings in specific directions and is compatible with scalable processing.^16,23–25,29–32^ The small interface area helps to limit the formation of interface related defects, and the shape of the nanostructure can be controlled by the geometry of the openings.^16,29,33,34^ While this helps minimise the interface related defects, there are other sources of defects in Zn_3_P_2_.^35–38^ Various defect levels in the bandgap have been experimentally probed by various groups previously, but very few have been able to describe the exact origins.^36,37,39–44^ Conversely, other studies have observed defects, such as rotated domains observed through electron microscopy, but their exact nature and influence on the materials properties is yet to be ascertained.^16^ This creates a need for systematic and comprehensive studies able to correlate the different factors related to defect structure and their influence in Zn_3_P_2_ in order for it to realise its potential.

An additional benefit of nanostructures is their nanophotonic properties, including directed emission, enhanced optical absorption cross-section, and light trapping to name a few.^45–49^ Nanophotonics make them ideal for optoelectronic and photovoltaic applications amongst other applications.^50–55^ The potential of nanostructured materials has been extensively researched for *e.g.* III–Vs, but their potential impact on for example earth-abundant II–V semiconductors such as Zn_3_P_2_ still remains to be fully explored.

To comprehensively understand the nanostructured material growth mechanisms with the aim to optimize its structure and properties, it is imperative to collect structural information down to the atomic level to accurately characterise material and defects. Currently, one of the sole techniques capable of such detailed characterisation is aberration-corrected scanning transmission electron microscopy (AC STEM). High-angle annular dark-field (HAADF) has been the dominating imaging mode due to its incoherent nature allowing direct imaging of atomic columns with elemental sensitivity and compatibility with a range of analytical techniques.^56–63^ Recent advances have increased the achievable resolution, allowing for the analysis of structural and chemical information down to the atomic level. Furthermore, HAADF-STEM opens the possibility to create 3D atomic models of the nanostructures under study, in order to get a deeper insight into the main growth processes from an atomistic point of view, and in parallel complement the study with DFT analysis for energetic calculations of the different interfaces created and a basic explanation of defect formation.^57,64,65^ Strain relaxation mechanism evaluation will follow by performing HAADF-STEM image simulation, from the 3D atomic models, and extended dedicated analysis such as geometrical phase analysis (GPA).^66–69^ The application of such technique to the experimental and simulated HAADF-STEM images ensures the possibility to verify the growth relaxation mechanisms involved in the nanostructure being studied, together with relative dilatation and rotation of the atomic planes, giving a complete illustration of the growth process characteristics.

Herein, we investigate SAE grown Zn_3_P_2_ nanowires through AC HAADF-STEM and simulations to elucidate the epitaxial relationship with the InP substrates and to gain insight into defect formation in the material and the potential influence of said defects on the final material performance.

## Results and discussion

**Fig. 1:**
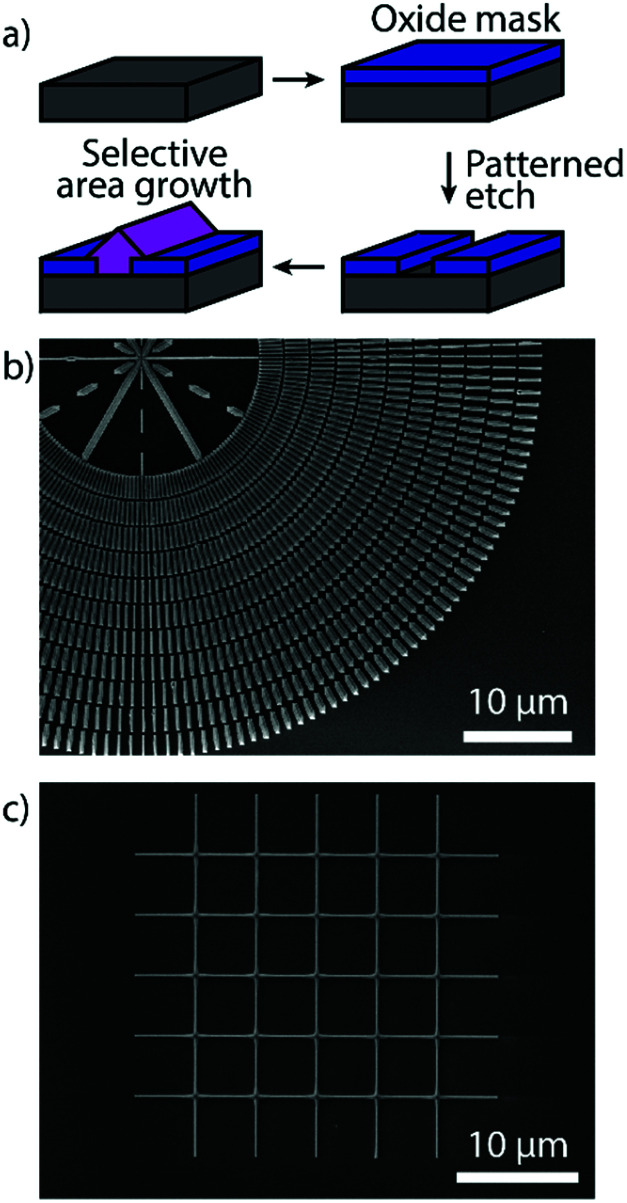
(a) Schematic of the SAE growth process for Zn_3_P_2_. (b) Overview SEM image of Zn_3_P_2_ nanowires grown at different angles by SAE. (c) SEM image of a Zn_3_P_2_ nanowire network grown by SAE.

**Fig. 2:**
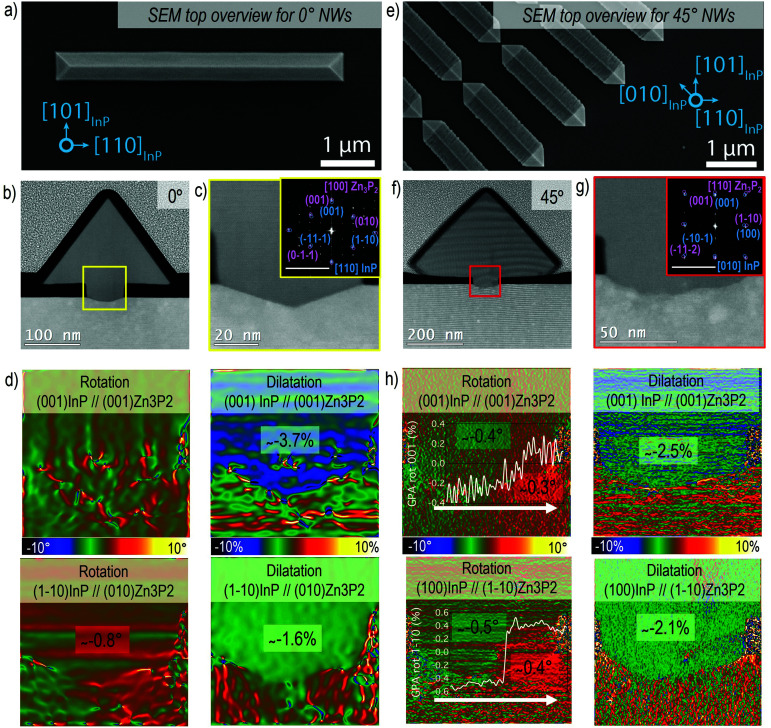
(a) SEM overview image showing the Zn_3_P_2_ nanowire morphology grown along <110> with respect to the InP substrate (0°). (b) HAADF-STEM overview image of the nanowire cross-section obtained from the nanowire in (a). (c) HAADF-STEM image of the detail in the nanowire-substrate interface area. The indexed power spectrum is reported in the inset to evaluate the mutual orientation of the two systems (scale bar 5 1 nm^−1^). (d) GPA rotational and dilatation maps in the interface area for the plane directions that are parallel and perpendicular to the interface. (e) SEM overview image showing the Zn_3_P_2_ nanowire morphology grown along <100> with respect to the InP substrate (45°). (f) HAADF-STEM overview image of the nanowire cross section obtained from the nanowire in (e). (g) HAADF-STEM image of the detail in the nanowire-substrate interface area. The indexed power spectrum is reported in the inset to evaluate the mutual orientation of the two systems (scale bar 5 1 nm^−1^). (h) GPA rotational and dilatation maps in the interface area for the plane directions that are parallel and perpendicular to the interface.

Horizontal Zn_3_P_2_ nanowires were grown by SAE through the scheme illustrated in Fig. 1a, showing how the grown material is localised to the exposed nanoscale holes (more details in the Methods section). By tuning the shape of the openings we can grow single-crystal nanowires of desired length at a range of different angles as shown in Fig. 1b. The material and interfacial quality remained unchanged irrespective of the angle as discussed in more detail below. We focused our attention on two crystalline orientations of the Zn_3_P_2_ nanowires which are at 45° with respect to each other. Specifically, the nanowires with their axial directions being (001) × [100]_Zn_3_P_2__ on (001) × [110]_InP_ (namely the 0° orientation) and (001) × [110]_Zn_3_P_2__ on (001) × [010]_InP_ (namely the 45° orientation). However, at intermediate angles with respect to the <110> directions of the InP (100) substrate the nanowires exhibit microfaceting leading to surface steps (see Fig. 2e). Furthermore, by patterning the openings into networks we were able to grow interconnected nanowires with regular junctions as shown in Fig. 1c, and even overgrow them into a textured film (Fig. S1†).

The crystal quality evaluation is summarised in Fig. 2 for horizontal nanowires at 0° (Fig. 2a–d) and 45° (Fig. 2e–h), respectively. Fig. 2a and e are SEM overview images of the nanowire. Here the [100]_Zn_3_P_2__ and [110]_Zn_3_P_2__ directions are parallel to [110]_InP_ and [010]_InP_, respectively, and the resulting nanowires grow epitaxially along the <001> direction. The nanowires present a triangular cross-sectional shape with the surface encompassed by the {011}_Zn_3_P_2__ and {0–11}_Zn_3_P_2__ family of planes. Considering the [100]_Zn_3_P_2__ direction parallel to [110]_InP_ (orientation 0°), we can observe that the interface between the substrate and the nanowire is sharp, as evidenced in Fig. 2b and c. The substrate presents some faceting inside the mask openings along the {1–13} planes, probably induced during the etching process. On the contrary, when [110]_Zn_3_P_2__ direction is parallel to [010]_InP_ (orientation 45°), the surface is rough as a consequence of microfaceting, as observed in Fig. 2f and g.

From the GPA analysis we could investigate plane relative distortions, in terms of dilatation and rotation, for the Zn_3_P_2_ nanowire with respect to the InP substrate, as reported in Fig. 2d and h. The residual strain is calculated considering the corresponding bulk material as in Fig. S2 and S3† and the results are summarised in Tables 1 and 2. In particular, we focused on the parallel and perpendicular directions with respect to the (001)_InP_ surface. When the [100]_Zn_3_P_2__ direction is parallel to [110]_InP_, the (001)_Zn_3_P_2__ (parallel) and (010)_Zn_3_P_2__ (perpendicular) planes are not significantly rotated with respect to the substrate, while in the 45° orientation there is a slight rotation towards positive angles following the white arrow in Fig. 2h, as indicated in the line profile in the inset, which previously has been attributed to strain relaxation mechanisms in SAE nanowires.^70^

**Table 1:** Summary of the plane mismatches for the 0° nanowire, specifically (001) × [100]_Zn_3_P_2__ on (001) × [110]_InP_

	Plane mismatch	
Direction	(001)_Zn_3_P_2__//(001)_InP_	(010)_Zn_3_P_2__//(1–10)_InP_
Measured (GPA)	−3.7%	−1.6%
Theory (bulk relaxed material)	−2.8%	−2.7%
Residual strain (nanowire)	−0.9%	+1.1%
	Compressive	Tensile

**Table 2:** Summary of the plane mismatches for the 45° nanowire, specifically (001) × [110]_Zn_3_P_2__ on (001) × [010]_InP_

	Plane Mismatch	
Direction	(001)_Zn_3_P_2__//(001)_InP_	(1–10)_Zn_3_P_2__//(100)_InP_
Measured (GPA)	−2.5%	−2.1%
Theory (bulk relaxed material)	−2.8%	−2.7%
Residual strain (nanowire)	+0.3%	+0.6%
	Tensile	Tensile

The material accommodation during the epitaxial growth is reflected in the overall plane dilatation in the nanowire. For the scenario when the [100]_Zn_3_P_2__ direction is parallel to [110]_InP_, the (001)_Zn_3_P_2__ planes present a 0.9% compression even after outgrowing the mask to accommodate the interfacial strain.^11,71^ Regarding the parallel planes, the (010)_Zn_3_P_2__, there is a remnant tensile strain causing a 1% expansion to ensure a better plane-to-plane adaptation at the interface and prevent the formation of misfit dislocations. These findings are in good agreement with the Zn_3_P_2_ pyramids grown on InP^16^ along the same orientation as for the 0° case, as reported in Fig. S4.† For the [110]_Zn_3_P_2__ direction parallel to [010]_InP_, the (001)_Zn_3_P_2__ planes exhibit almost complete relaxation, while the (1–10)_Zn_3_P_2__ planes are slightly expanded (tensile strain).

Therefore, for both investigated orientations, there is a small component of tensile strain along the <1–10>_Zn_3_P_2__ direction that results in a defect-free epitaxial relationship between the Zn_3_P_2_ nanowire's lattice with respect to the InP substrate. In fact, the remnant strains for the <1–10>_Zn_3_P_2__ nanowires are actually lower than those of the <100>_Zn_3_P_2__ nanowires. From this analysis we can conclude that the SAE approach is successful in terms of high crystal and interface quality. The resulting Zn_3_P_2_ is minimally strained with respect to its bulk counterpart, preventing the formation of misfit dislocations and strain build-up that could cause crack formation, both which are detrimental for the final material's application performance. Further details on the GPA analyses have been reported in the ESI.†

**Fig. 3:**
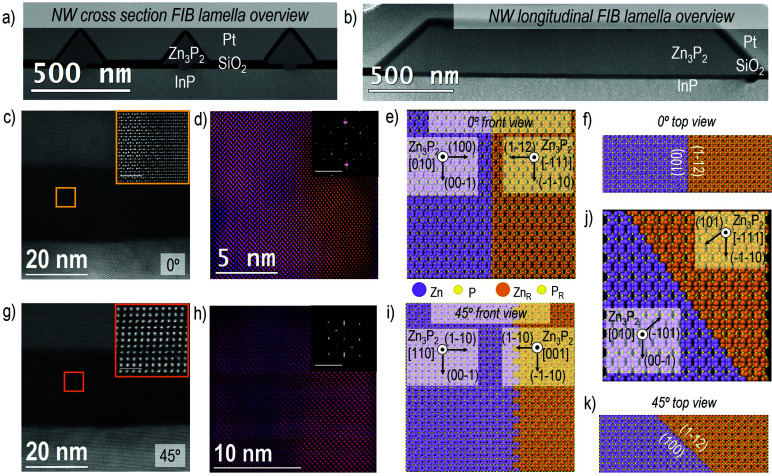
(a) HAADF-STEM overview image of the cross-sectional FIB lamella. (b) HAADF-STEM overview image of the longitudinal FIB lamella. (c) HAADF-STEM image of the detail in the nanowire-substrate interface area for 0° orientation. In the inset the high-magnification image is reported (scale bar 1 nm). (d) Frequency filtered map of the inset in (c) showing the presence of two Zn_3_P_2_ main domains with different colour: [010]_Zn_3_P_2__ in violet and [111]_Zn_3_P_2__ in orange. In the inset, the power spectra for the two domains are reported with different reflections corresponding to each domain. 3D atomic model of the different domain interface from front and top view are shown in (e) and (f), respectively. (g) HAADF-STEM image of the nanowire-substrate interface area for 45° orientation. In the inset a high-magnification image is included (scale bar 1 nm). (h) Frequency filtered map of the inset image in (c) highlighting the presence of two Zn_3_P_2_ rotated domains with different colour: [001]_Zn_3_P_2__ in orange and [110]_Zn_3_P_2__ in violet. In the inset the power spectra for the two domains are reported indicating different reflections corresponding to each domain (scale bar 2 1 nm^−1^). 3D atomic models of the domain interface from the front (i) and top (k). (j) Shows a 3D atomic model of the (101) interface of two domains rotated 120° with respect to the interface.

In Fig. 3 we report some examples of another type of defect in the Zn_3_P_2_ nanowires that may be more difficult to control during growth, which is the presence of different rotated domains within the length of the nanowire. Fig. 3a and b show a general overview of the obtained FIB lamellae for both the cross-section (Fig. 3a) and longitudinal (Fig. 3b) cuts. It is possible to recognize the presence of more than one domain already from the HAADF-STEM image contrast (as in the insets of Fig. 3c and g) and/or corresponding power spectrum analysis. The different Zn_3_P_2_ crystal domains observed in a nanowire with a <010>_Zn_3_P_2__ axial direction are highlighted in Fig. 3d in the frequency filtered map, obtained from the Zn_3_P_2_ inside the SiO_2_ mask, as indicated with the yellow square in Fig. 3c. In the inset of Fig. 3d we show the power spectra in the region of interest where the colour of the spectra corresponds to the regions in the filtered map on the same zone axis. In the orange marked area, the Zn_3_P_2_ is observed along its [−111] zone axis, while in the violet area the Zn_3_P_2_ is found rotated to its [010] zone axis. As can be seen from the simulated diffraction patterns, the main reflections arising from [−111] zone axis perfectly overlap with some of the reflections arising from the [010] zone axis. Therefore, while it is straightforward to filter and show the [010] oriented domains thanks to their extra diffraction spots or frequencies, it is challenging to separate the [−111] oriented domain as every diffraction spot here has a similar frequency for the other orientation. Furthermore, in both domains the atomic arrangement is similar, therefore just residual strain and no presence of dislocations have been detected *via* GPA analysis. Zn_3_P_2_ tends to form these two stable rotated domains already in the growth region confined by the SiO_2_ mask apertures, and they extend even further (see more images in Fig. S5†). Nonetheless, in all the multidomain defects observed for the 0° case, the interface between the two domains is sharp and parallel to the zone axis, as highlighted in the representative model reported in Fig. 3e. The crystallographic directions for the two domains are reported, together with the top view (in Fig. 3f) highlighting the presence of a sharp boundary along (010) × [100]_Zn_3_P_2__ (main) and (1–12) × [111]_Zn_3_P_2__ (rotated), interfacing the different domain orientations.

**Fig. 4:**
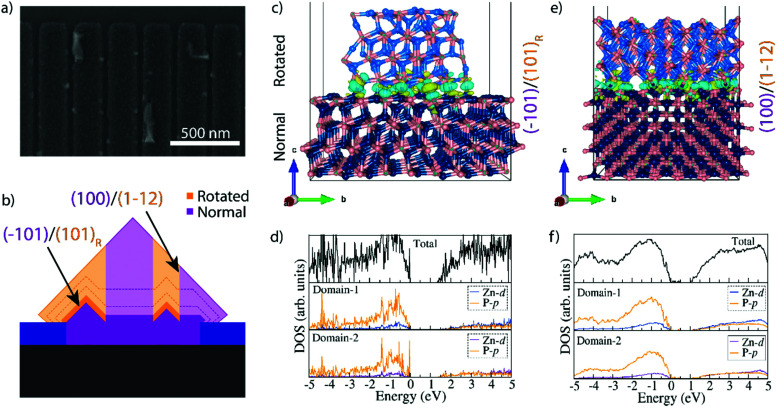
(a) SEM image showing the formation of (101) faceted domains within the holes. (b) Diagram showing the proposed change in growth direction when the growth occurs on (001) *vs*. (101) facets. (c) Atomic positions and electron distribution as used for the DFT calculations of two domains joined with a (101) plane and the upper domain rotated 120°, resulting in a stable interface without dangling bonds. (d) Density of states at the interface showing an overall decrease of the bandgap from 1.50 eV to 1.31–1.33 eV while no interband states are observed. (e) Atomic positions and electron distribution as used for the DFT calculations of two domains rotated 120° around the (101) plane and joined by (100) and (1–12) facets. (f) Density of states at the interface showing an overall decrease of the bandgap from 1.50 eV to 1.02 eV and low density of interband states at energies of 0.10 eV, 0.21 eV, and 0.38 eV above the valence band.

Delving deeper into the 45° case, the observed domains are similarly highlighted in the frequency filtered map in Fig. 3h, obtained from the Zn_3_P_2_ inside the SiO_2_ mask as indicated in the orange square in Fig. 3g. As previously shown, both the frequency filtered map and the power spectra show clearly the presence of rotated domains which are specifically oriented along [110]_Zn_3_P_2__ (orange region) and [001]_Zn_3_P_2__ (violet region) zone axes. In this case, the boundaries that separate the two domains are not parallel to the zone axis, as for the 0° nanowires. Taking into account both, the image contrast and power spectrum analysis, here we propose that the domain boundary is transversal (45°) with respect to the zone axis, generating a discontinuity in the structure that forms the two domains with orientations equivalent to [110] × (001) and [001] × (110), as it is possible to observe in the model and corresponding orientation in Fig. 3i. Fig. 3k shows a top view of the domain boundary, highlighting its orientation. The exact origin of these rotated domains is discussed in more detail below, but is believed to be connected to the switching of the growth front to {101}_Zn_3_P_2__ surfaces, so for a complete picture we also included this combination of rotated {101}_Zn_3_P_2__ facets in Fig. 3j. Although this combination remains a small proportion as it is only related to the nucleation site, and other interfaces are formed during growth (see Fig. 4b).

It is possible to observe that for 0° and 45° orientations, the majority of the Zn_3_P_2_ domains exhibit a <001>_Zn_3_P_2__ direction perpendicular to the substrate, while the rotated domain (<110>_Zn_3_P_2__ direction perpendicular to the substrate) appears with a lower density all along the volume of the nanowire (see Fig. S5†). Animated movies of the rotational domain formation for different nanowire orientations can be found in the following: movie 1 (0° NW)^90^ and movie 2 (45° NW).^91^ In order to further investigate and understand this, we reproduced all the observed domains with 3D atomic models, with special attention on the Zn_3_P_2_–InP interface. By performing image simulation and GPA analysis we could observe that out of the two observed orientations, the domains with [001] as growth direction are the most favorable in terms of mismatch (as reported in Fig. S6†).

A previous study observing these domains in Zn_3_P_2_ nanopyramids argued that the formation of the rotated domains was related to the growth plane switching from (001) to (101), as it was then only observed when it switched from axial to lateral growth.^16^ However, we observe them within the nanowires themselves. We thus dwelled in the early stages of growth for a further understanding how the nanowire form. Fig. 4a shows a representative SEM micrograph of a sample grown for 15 minutes. We observe that small domains with what appears to be (101) facets have started to form. These domains are randomly distributed within the localized growth area, but they all seem to be at least partially touching the edge of the holes (Fig. S7†). These domains thus provide the (101) facets for simultaneous growth of regions with different rotation. This mechanism is illustrated in Fig. 4b. We propose that the domains of different orientation will compete during the selective area growth, till they are completely blended, forming a nanofaceted nanowire structure with the buried interfaces.

In the hypothesis that the rotation would be strictly related to growth on (101) facets, one should also observe how they rotate again eventually. However, this is not observed. In order to gain a deeper insight into the stability of these domains we turned to DFT calculations and investigated the difference in energetics and electronic structure when joining two Zn_3_P_2_ domains at (101) facets as a function of the rotation (0° or 120°), as shown in Fig. 4c, d and Fig. S8.†Fig. 4c shows the configuration observed when two rotated domains (120°) with relaxed (101) surfaces are brought into contact. Interestingly, we observe that there should not be any dangling bonds at the interface. Furthermore, the calculated interface adhesive energy for this scenario (*E*_ad_) is found to be −0.179 eV. When using non-relaxed surfaces, we observed slight changes in the configuration, however, we did not observe any dangling bonds and *E*_ad_ for this scenario was −0.167 eV (Fig. S8†). The final scenario we simulated for comparison was the joining of two non-rotated domains. As expected, there were no dangling bonds formed and it is interesting to highlight that the calculated *E*_ad_ is comparable to the other scenarios, namely −0.175 eV. In fact, all these values are within the approach's error of each other. This has two consequences: (i) differences due to the use of relaxed or non-relaxed surfaces in this case are negligible, and (ii) there is no significant penalty for forming a rotated interface and they exhibit a similar stability to a non-rotated interface. In order to further elucidate the exact conditions needed for the rotation to occur one would need to include the kinetics and growth rates, through for example phase-field modelling. This could potentially show how growth conditions and parameters could be optimised to minimise these domains, but this is beyond the scope of this study. As the domains grow they will also form extended (100)_Zn_3_P_2__//(112)_Zn_3_P_2__ interfaces between these domains. This combination also has a very low *E*_ad_ (−0.203 eV) as calculated by DFT, indicative of a high stability.

Another interesting observation from the DFT results is the change in electronic structure around the interfaces of the rotated domains as shown in Fig. 4d and Fig. S8.† The first thing to note is that there are no states within the bandgap for the (101) interface, however, the (100)_Zn_3_P_2__/(112)_Zn_3_P_2__ case did show a low density of shallow states at energies of 0.10 eV, 0.21 eV and 0.38 eV above the valence band. Moreover, we do observe a decrease in the overall bandgap. Compared to the bulk material where a DFT+U approach predicted the band gap at 1.44 eV, a slight underestimation relative the HSE06 functional (1.51 eV), the DFT+U estimated bandgap was 1.33 eV when relaxed surfaces were joined (1.31 eV for non-relaxed). A less significant shrinkage (1.37 eV) was observed for the non-rotated domains. In the (100)_Zn_3_P_2__//(112)_Zn_3_P_2__ case the shrinkage was even greater, resulting in a bandgap of 1.02 eV. Due to large number of atoms (640 in total) in the interface system, the HSE06 functional could not be used. While the accuracy of the DFT+U approach is not sufficient to establish the exact energies, the overall trend still stands and is of great interest. Previous experimental studies of Zn_3_P_2_, including SAE grown Zn_3_P_2_,^16^ have shown transitions or defect levels in the range of 0.14–0.20 eV, 0.25–0.29 eV, and 0.36 eV below the bandgap, which puts them in the potential range of these rotated interfaces.^6,16–18,36,37,39–44,72^ While there may be other origins of transitions in this range acting in parallel, these rotated domains are a potential source. With this in mind, we inspected the surface of zigzag nanowires grown by a vapour–liquid–solid (VLS) growth mechanism (as opposed to the vapour–solid (VS) one of SAE) that are terminated with (101) facets, but still exhibit sub-bandgap emission in a similar range as to those calculated here.^14,15,17,72^ The VLS grown nanowires did contain small, rotated domains on the surface in crevices with an increased portion of radial VS growth (Fig. S9†). No domains were observed in the VLS grown cores. These domains can therefore be attributed as a potential source of the sub-bandgap emission observed from these structures, however, the spatial resolution used in these studies was not sufficient to accurately attribute this emission solely to these surface domains.^17,72^

## Conclusions

Selective area epitaxy has been shown herein as a promising approach to produce high-quality horizontal nanowires from the earth-abundant compound semiconductor Zn_3_P_2_ in various complex configurations. To properly assess these structures, we turned to AC HAADF-STEM, combined with 3D atomic modelling and image simulation, which showed that there were no misfit dislocations forming at the interface, and that all strain was accommodated elastically. The vertical growth direction was determined to be [001], which is the ideal growth direction to minimize interfacial strain. In addition, we were able to observe domains rotated 120° within the entire nanowires. These domains were formed when growth switched from (001)_Zn_3_P_2__ facets to (101)_Zn_3_P_2__ ones. Further growth of the rotated domains resulted in extended (100)_Zn_3_P_2__//(112)_Zn_3_P_2__ interfaces. DFT calculations showed that the domain boundaries associated with this rotation are very stable. Furthermore, we have been unable to observe a rotation back to the original orientation, indicating that the initial rotation is a consequence of a change in growth kinetics related to the formation or disappearance of competing surfaces. Finally, through the DFT calculations we could determine that no dangling bonds or mid-gap states are formed at the rotated (101) interface, albeit the (100)_Zn_3_P_2__//(112)_Zn_3_P_2__ interfaces do exhibit shallow states at 0.10 eV, 0.21 eV, and 0.38 eV according to the DFT calculations. In addition, we observe a shrinkage of the bandgap to approximately 1.31–1.33 eV for the (101) interface and 1.02 eV for the (100)_Zn_3_P_2__//(112)_Zn_3_P_2__ case. While there is still uncertainty associated with the energy values calculated through DFT, this could potentially explain the transitions observed in this energy range by previous studies. This study is a significant step towards the utilisation of selective area epitaxy to grow complex lattice-mismatched structures outside of III–V materials and highlighting the capabilities of combining AC HAADF-STEM and DFT to fully understand the defect formation and impact in novel materials.

## Methods

The 30 nm thick SiO_2_ mask layer was deposited using plasma-enhanced chemical vapour deposition (PECVD, Oxford Plasmalab System100) on InP(100) *epi*-ready substrates. Next, the pattern was created using electron-beam lithography (Raith EBPG5000+) and etching using a combination of dry (fluorine plasma, SPTS APS) and a wet etch (BHF final dip for 10s). The samples were then transferred to a Veeco GENxplor molecular beam epitaxy system, where the substrates were fist degassed for 1.5 h at 150 °C and 300 °C for 2 h before being introduced into the growth chamber. Additional growth details can be found in ref. 16.

TEM samples were prepared using focused ion beam (FIB) processing. Before the samples were processed, a 20 nm thick SiO_2_ film was deposited using PECVD. A Focused Ion Beam HELIOS 600 FIB system was then used to create electron transparent lamellae of longitudinal and transverse sections of the nanowires growing along the [110] (0°) and [010] (45°) of the InP substrate. Aberration corrected HAADF-STEM was carried out using an FEI Titan probe corrected transmission electron microscope. Electron Energy Loss Spectroscopy in STEM mode (EELS-STEM) compositional maps have been obtained in the in a Tecnai F20 microscope by using a GATAN QUANTUM filter. 3D atomic models have been created by using Rhodius^73,74^ and STEM_CELL softwares,^75–77^ the latter was also used for STEM image simulation. Geometrical phase analysis (GPA) has been performed on simulated and experimental images by using the licenced GPA plug-in available in Gatan Digital Micrograph.

The density functional theory (DFT) calculations were performed within periodic boundary conditions using the Vienna Ab Initio Simulation Package (VASP).^78–80^ The projected augmented wave (PAW) method^81^ was used to describe the interactions between the valence and cores electrons. The Perdew–Burke–Ernzerhof (PBE) generalized gradient approximation (GGA) functional was used to calculate electronic exchange–correlation potential.^82^ A plane wave cutoff energy of 600 eV and a Gamma-centred *k*-point mesh of 5 × 5 × 3 were found to converge the total energy of Zn_3_P_2_ to within 10^−6^ eV and the residual Hellmann–Feynman forces on all relaxed atoms reached 0.01 eV Å^−1^. A full unit cell relaxation yielded a strain-free Zn_3_P_2_ with lattice parameters *a* = *b* = 8.029 Å, *c* = 11.336 Å, which compares closely with available experimental data. Given the fact that the 3d-electrons of transition metals are strongly correlated, methods beyond standard density functional theory (DFT) are needed to correctly describe transition metal-based materials in terms of electronic and magnetic properties.^83–85^ To accurately predict the electronic bandgap of Zn_3_P_2_, we have used both hybrid HSE06 functional with 25% Hartree–Fock exchange^86^ and Hubbard correction (DFT+U)^87^ with *U*_eff_ value of 20 eV found for Zn ions. The hybrid HSE06 functional gave a bandgap of 1.51 eV good agreement with experiment whiles the DFT+U approach slightly underestimates the bandgap at 1.44 eV. The DFT+U correction approach adds an on-site Coulomb repulsion to the DFT Hamiltonian, providing a better treatment of the strong electron correlation in the localized Zn d-orbitals. Thus it predicts the electronic band gap of Zn_3_P_2_ in closer agreement with the hybrid HSE06 functional compared to the standard DFT-PBE functional, which significantly underestimates the band gap at 0.67 eV. The METADISE code,^88^ was used to create the (101), (100), and (112) surfaces from the fully relaxed bulk material. To evaluate the interface energetic and properties of domains formed with the (101) facets joined together such the upper domain is rotated 120°, a supercell approach was employed. For the (101) interface, the bottom domain was constructed with (3 × 2)–(101) units, which is large enough to accommodate the growth of the 120° rotated top domain constructed with (2 × 1)–(101) units. For the (100)_Zn_3_P_2__/(112)_Zn_3_P_2__ interface, the bottom ((100)_Zn_3_P_2__) and top ((112)_Zn_3_P_2__) domains were constructed with (3 × 2) and (2 × 2) unit, respectively. Due to large unit cell size and number of atoms in the interface system (640 for the (101) interface and 1200 for the (100)_Zn_3_P_2__/(112)_Zn_3_P_2__ interface), only a 1 × 1 × 1 mesh of *k*-point was use for geometry optimisation. This was, however, increased to 3 × 3 × 1 for accurate determination of the electronic structure where the DFT+U approach was employed. The thermodynamic stability of the domain interface was evaluated *via* interfacial adhesion energy, calculated as: *E*_ad_ = (*E*_domain1/domain2_ − (*E*_domain1_ + *E*_domain2_)/*S*, where *E*_*domain1*/domain2_ is the total relaxed energies of the bottom/top domain interface structure with interface surface area *S*.^89^*E*_domain1_ and *E*_domain2_ represent the individual ground state relaxed total energy of the bottom (domain1) and top (domain2), respectively.

## Conflicts of interest

There are no conflicts to declare.

## Supplementary Material

NR-013-D1NR06190A-s001
