# Supplementary material for: Rotated domains in selective area epitaxy grown Zn3P2: formation mechanism and functionality
Source: Nanoscale. 2021 Oct 30;13(44):18441–50. doi: 10.1039/d1nr06190a (PMC8900489; doi:10.1039/d1nr06190a)
Supplement: NR-013-D1NR06190A-s001 [file NR-013-D1NR06190A-s001.pdf]

Electronic Supplementary Material (ESI) for Nanoscale.
This journal is © The Royal Society of Chemistry 2021

Supporting information to:

Rotated domains in selective area epitaxy grown Zn_3P_2: formation mechanism and functionality

Maria Chiara Spadaro[1#], Simon Escobar Steinvall[2#°], Nelson Y. Dzade[3,4], Sara Martí-Sánchez[1], Pol Torres-Vila[1], Elias Z. Stutz[2], Mahdi Zamani[2], Rajrupa Paul[2], Jean-Baptiste Leran[2], Anna Fontcuberta i Morral[2,5*], Jordi Arbiol[1,6*]

1. Catalan Institute of Nanoscience and Nanotechnology (ICN2), CSIC and BIST, Campus UAB, Bellaterra, Barcelona, Catalonia, 08193, Spain
2. Laboratory of Semiconductor Materials, Institute of Materials, Ecole Polytechnique Fédérale de Lausanne, 1015 Lausanne, Switzerland
3. School of Chemistry, Cardiff University, Main Building, Park Place, CF10 3AT Cardiff, UK
4. Department of Energy and Mineral Engineering, Pennsylvania State University, University Park, PA 16802, United States
5. Institute of Physics, Ecole Polytechnique Fédérale de Lausanne, 1015 Lausanne, Switzerland
6. ICREA, Pg. Lluís Companys 23, 08010, Barcelona, Catalonia, Spain

° Current affiliation: Center for Analysis and Synthesis and NanoLund, Lund University, Box 124, 221 00 Lund, Sweden

[#]These authors contributed equally to this work

Email: arbiol@icrea.cat; anna.fontcuberta-morral@epfl.ch

In Figure S1 we report an SEM overview image of different horizontal nanowires that coalesce together creating a textured film.

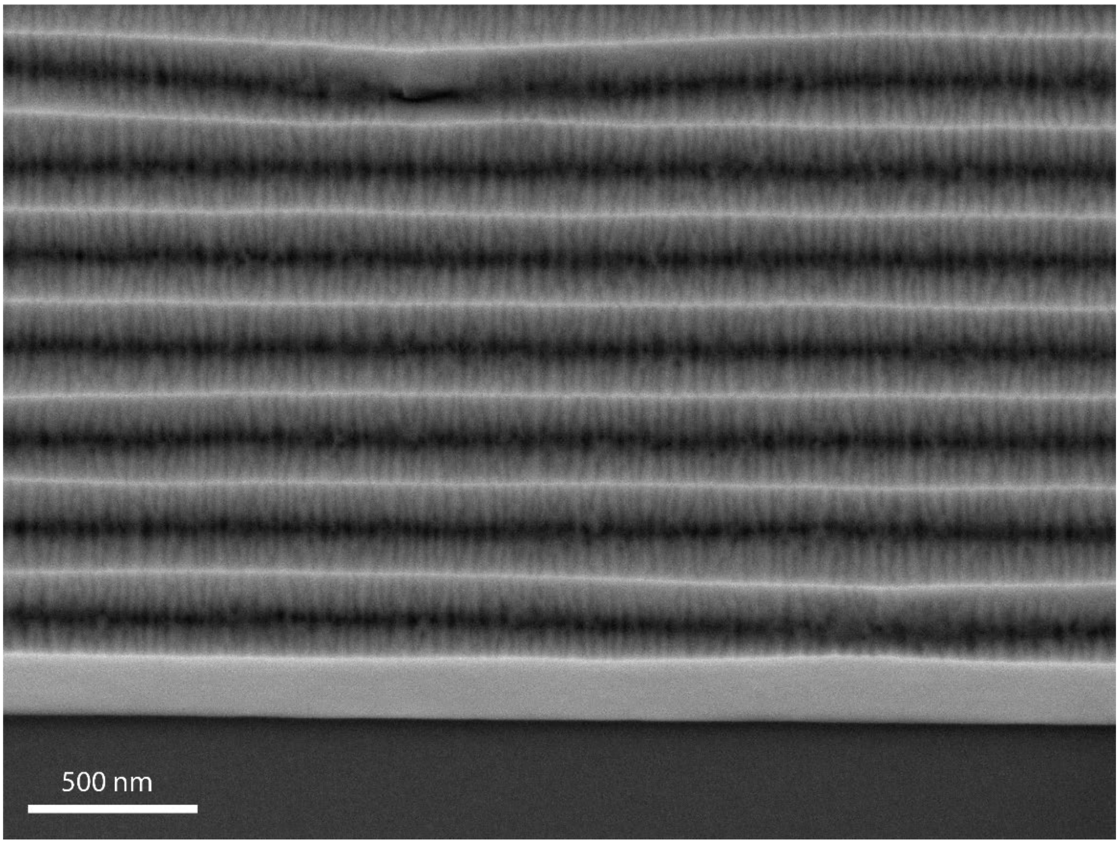

Figure S1. SEM image of horizontal nanowires coalescing into a textured film.

In Figures S2 and S3 we report the 3D atomic model, HAADF-STEM image simulation and GPA analysis for the nanowire-substrate interface in the two orientation of interest; the $[100]_{Zn3P2}$ and $[110]_{Zn3P2}$ directions are parallel to $[110]_{InP}$ and $[010]_{InP}$, respectively. The results here reported are used to evaluate the residual strain in the material.

For the $[100]_{Zn3P2}$ direction parallel to $[110]_{InP}$, if we consider the vertical direction to the substrate surface, we measure a -3.7% relative mismatch for the $(001)_{Zn3P2}$ plane with respect to the $(001)_{InP}$ plane (Figure 2d). For a fully relaxed Zn_3P_2 structure (as in Figure S2), we would expect -2.8% relative mismatch. Regarding the parallel planes to the $(001)_{InP}$ surface, the $(010)_{Zn3P2}$, a fully relaxed material should show a -2.7% relative lattice mismatch with respect to the $(1\text{-}10)_{InP}$ planes of the substrate (see image simulation in Figure S2).[11,70] In these nanowires, the $(010)_{Zn3P2}$ planes are only decreasing the mismatch by -1.6% (Figure 2d),

When the $[110]_{Zn3P2}$ direction parallel to $[010]_{InP}$, from the dilatation map of the planes parallel to the $(001)_{InP}$ surface we can observe that the lattice distance of the $(001)_{Zn3P2}$ planes is around 2.5% smaller than that of the $(001)_{InP}$ planes Figure 2h), as expected for a mostly relaxed Zn_3P_2 structure (in absence of strain the expected mismatch is -2.8%, as reported in the image simulation in Figure S3), as $d_{(004)_{Zn3P2}}$ = 0.2849 nm and $d_{(002)_{InP}}$ = 0.2934 nm.[11,12] Considering the planes perpendicular to the $(001)_{InP}$ surface, from the dilatation map we can observe that the lattice distance for the $(1\text{-}10)_{Zn3P2}$ planes is around 2.1% smaller (-2.1% mismatch) than that of the $(100)_{InP}$ planes in the substrate (Figure 2h), as expected for a slightly expanded Zn_3P_2 structure (in absence of strain the expected mismatch is -2.7% as reported in the image simulation in the Figure S3). The interplanar spacings according to literature are $d_{(2-20)_{Zn3P2}} = 0.2856$ nm and $d_{(200)_{InP}} = 0.2934$ nm, respectively.[11,70]

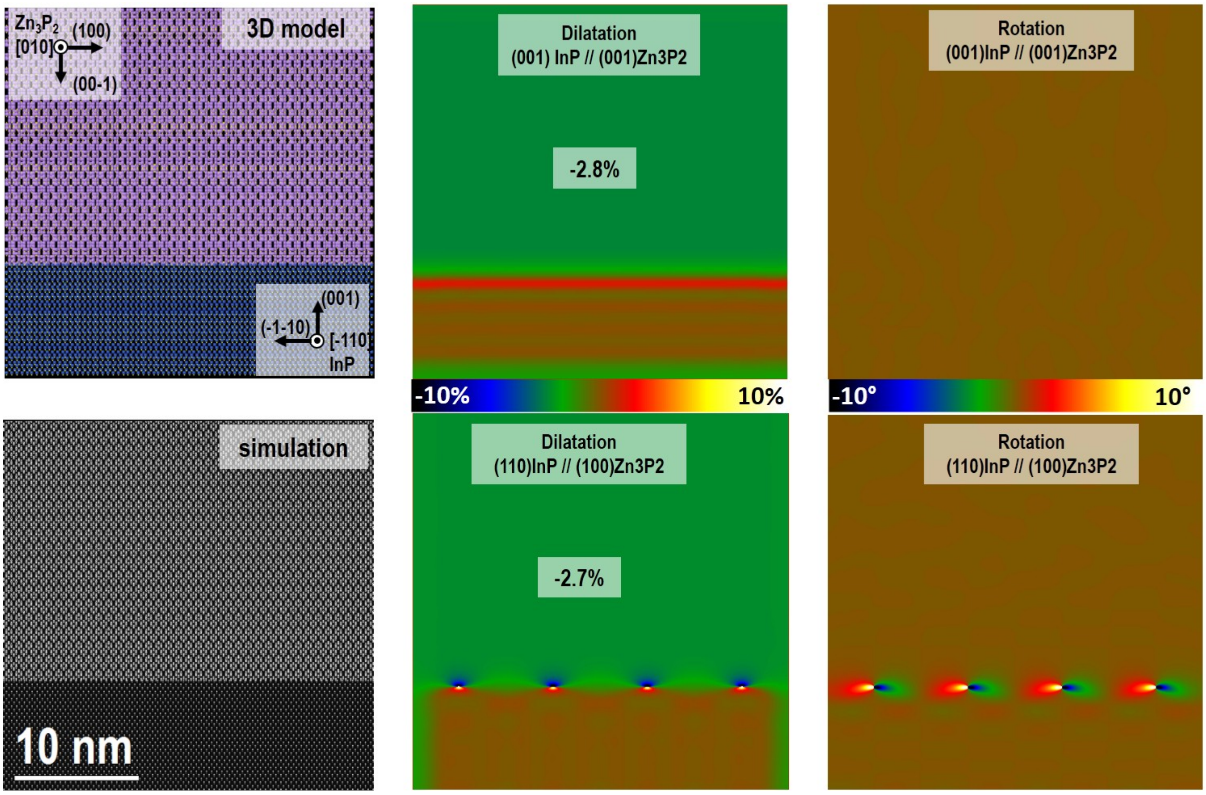

Figure S2. (left panel) 3D atomic and corresponding STEM image simulation for the (001)x[100]$_{Zn3P2}$ on (001)x[110]$_{InP}$. In the central and right panels the GPA analysis along the parallel (top panel) and perpendicular direction (bottom panel) with respect to the growth (001) direction.

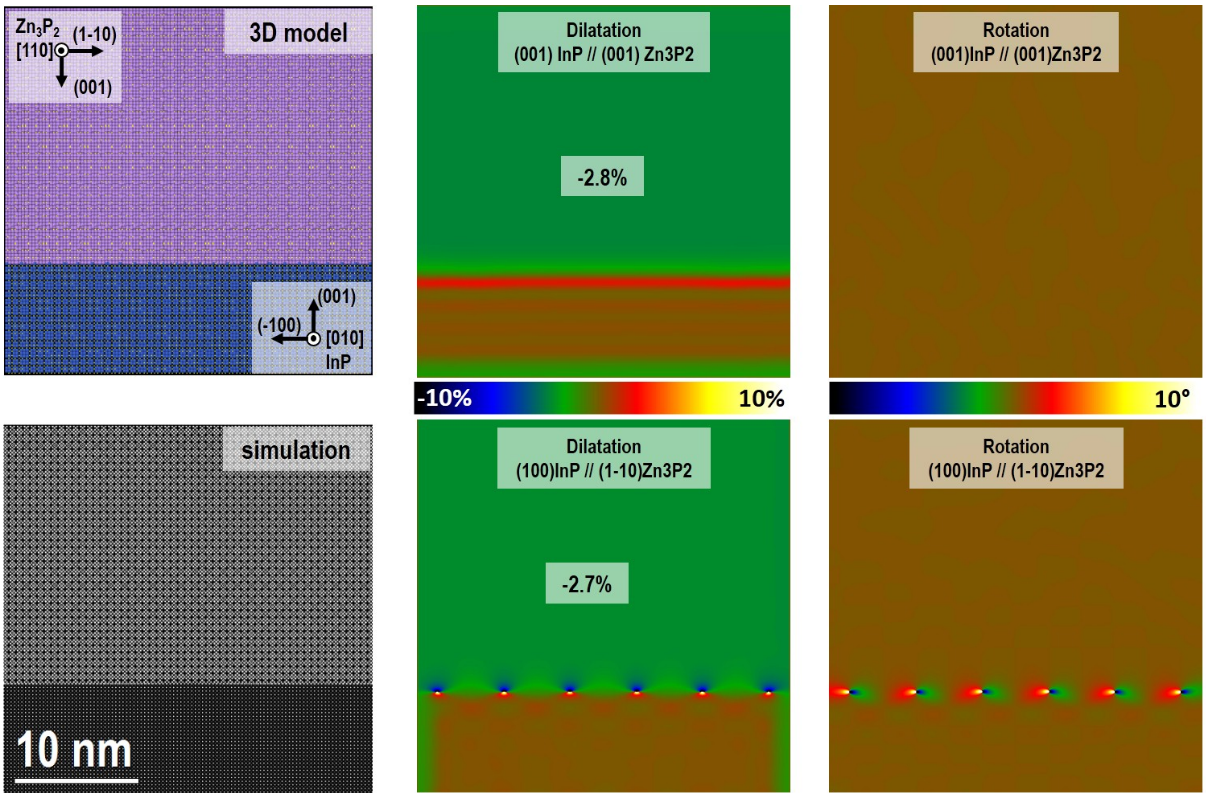

Figure S3. (left panel) 3D atomic and corresponding STEM image simulation for the (001)x[110]$_{Zn3P2}$ on (001)x[010]$_{InP}$. In the central and right panels the GPA analysis along the parallel (top panel) and perpendicular direction (bottom panel) with respect to the growth (001) direction.

In Figure S4 we report the HAADF-STEM image and GPA analysis on Zn3P2 nanopyramids grown on InP. By considering the perpendicular and parallel direction with respect to the substrate surface, we could observe -3.2% and -1.9% decrease on the lattice mismatch, respectively, and negligible rotation.

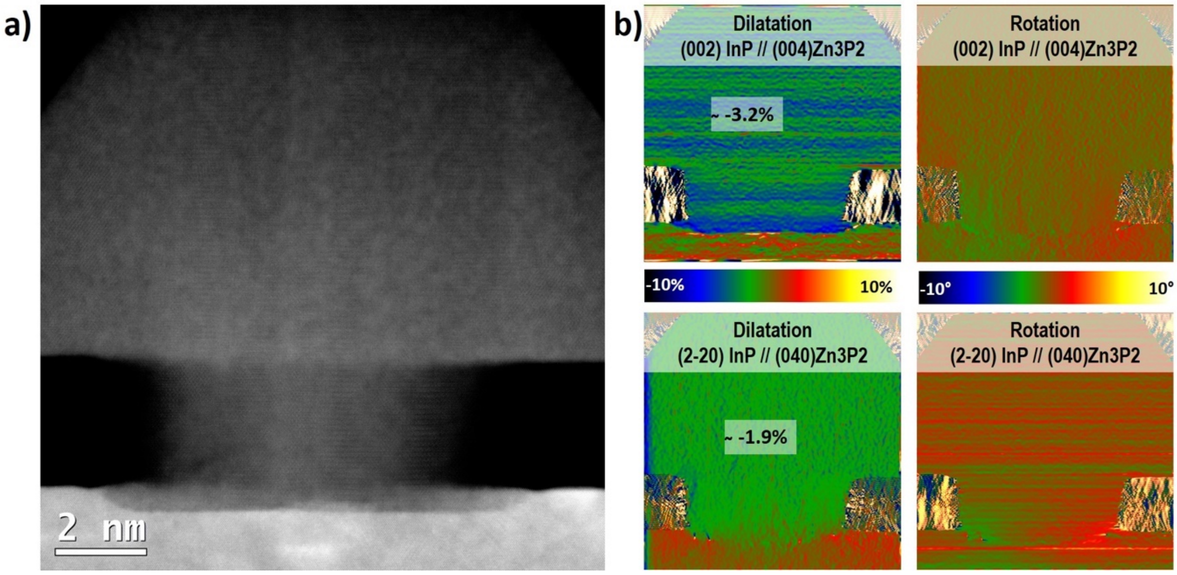

Figure S4. a) HAADF-STEM micrograph from Zn_3P_2 pyramid growing on InP substrate imaged along the $[100]_{Zn3P2}$ and $[110]_{InP}$ zone axis. b) GPA analysis along the parallel (top panel) and perpendicular direction (bottom panel) with respect to the growth (001) direction.

In Figure S5 we report some examples for the presence of the rotated domains along the investigated nanowires.

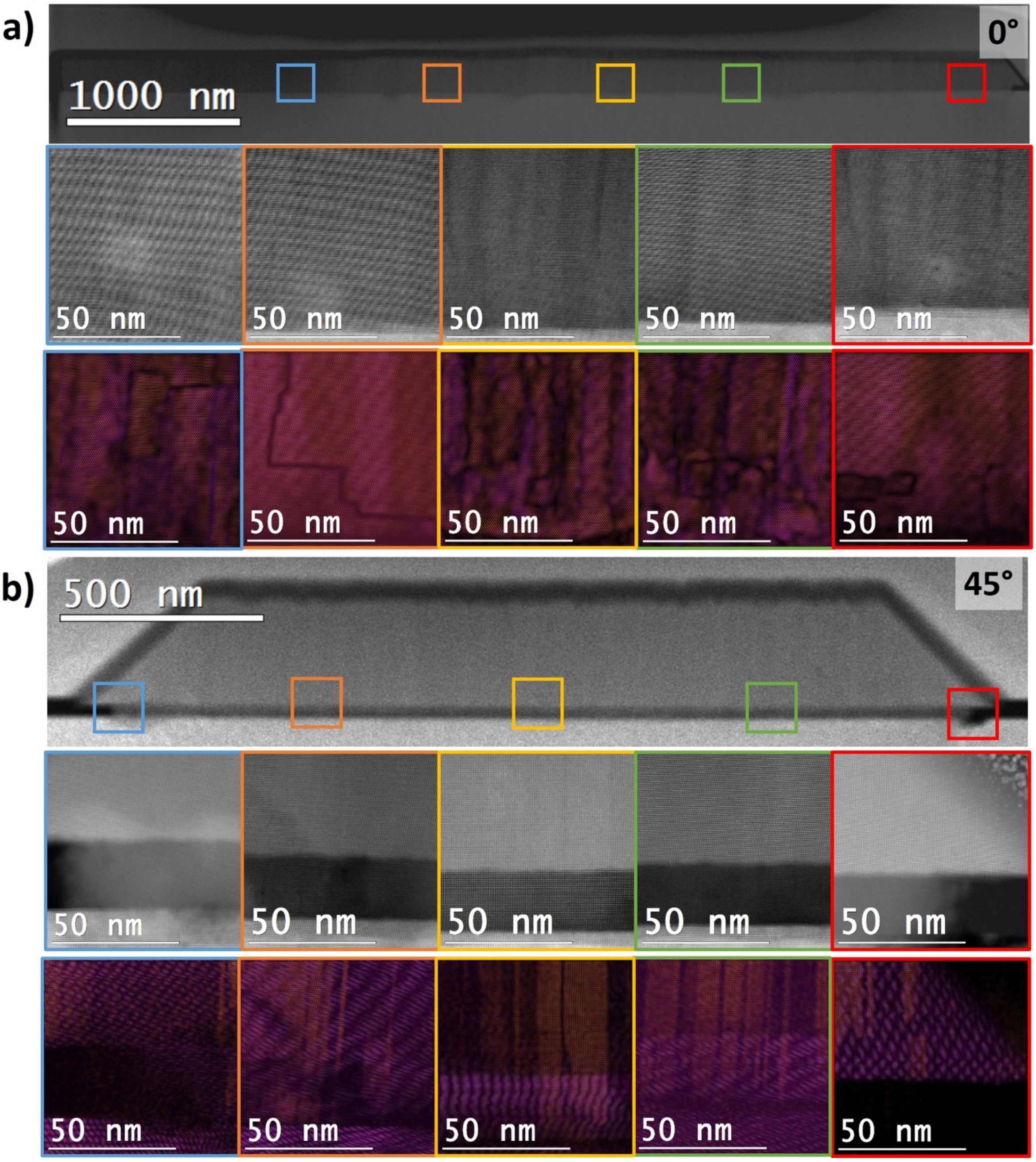

Figure S5. a) STEM-HAADF micrograph overview of the 0° nanowire. Here different areas are highlighted with coloured boxes; in the second and third panel the highlighted areas are reported with higher resolution together with the frequency filtered maps in order to highlight the presence of the two domains. b) STEM-HAADF micrograph overview of the 45° nanowire. Here different areas are highlighted with coloured boxes; in the fifth and sixth panel the highlighted areas are reported with higher resolution together with the frequency filtered maps in order to highlight the presence of the two domains.

In Figure S6 we report the 3D atomic model, HAADF-STEM image simulation and GPA analysis for the nanowire-substrate interface for both the main and rotated domains observed in the two orientations of interest; the $[100]_{Zn3P2}$ and $[110]_{Zn3P2}$ directions are parallel to $[110]_{InP}$ and $[010]_{InP}$, respectively.

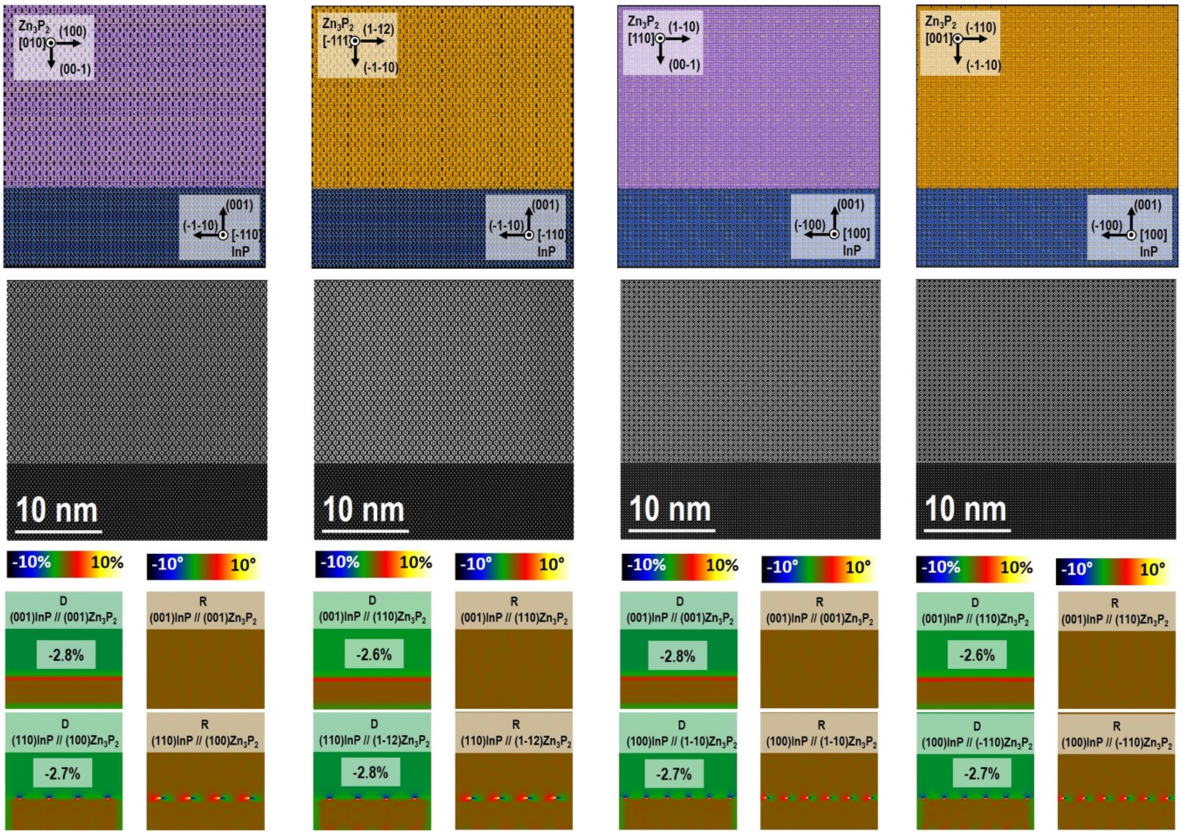

Figure S6. (top and central panels) 3D atomic and corresponding STEM image simulation for the main and rotated domains observed in the nanowire. In the bottom panel, the GPA analysis along the parallel (top panel) and perpendicular direction (bottom panel) with respect to the growth/vertical direction.

In Figure S7 we report the presence of the early stage (101) faceted domain nucleation along the entire nanowire.

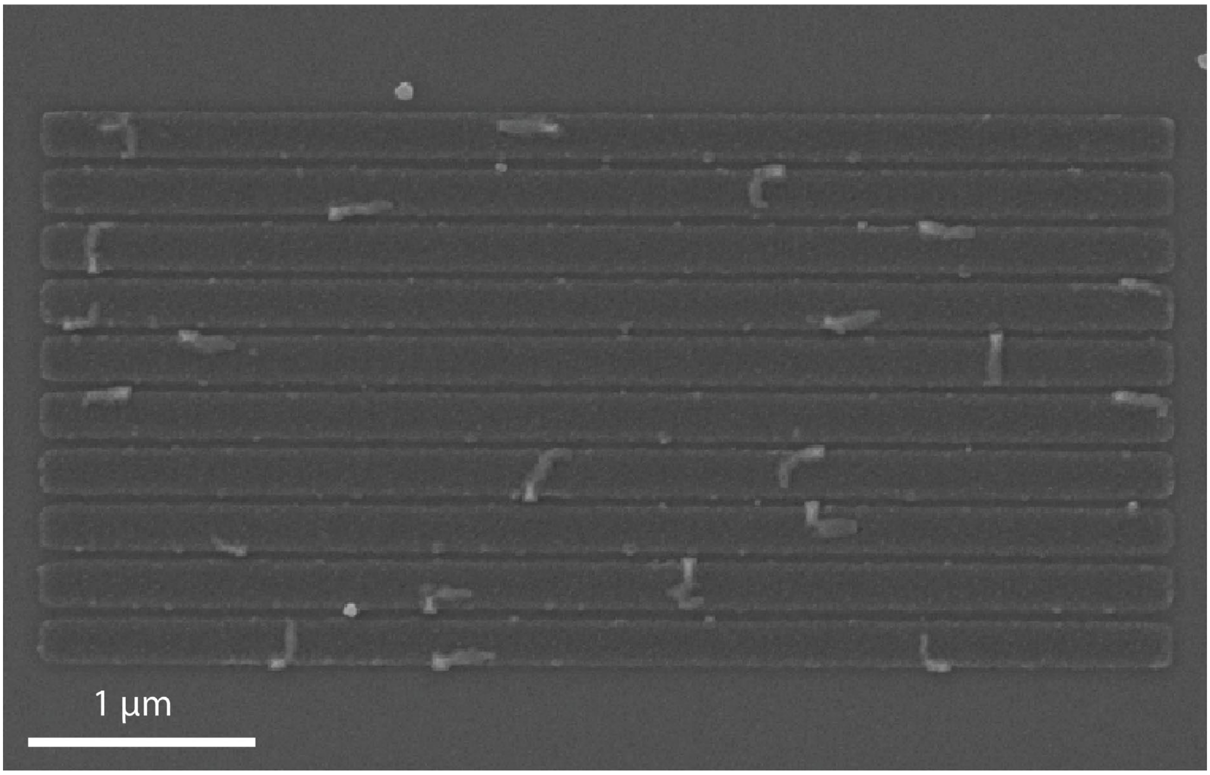

Figure S7. SEM image showing distribution of early stage (101) faceted domain nucleation.

In Figure S8 some extra DFT simulation results for the investigated interfaces are reported.

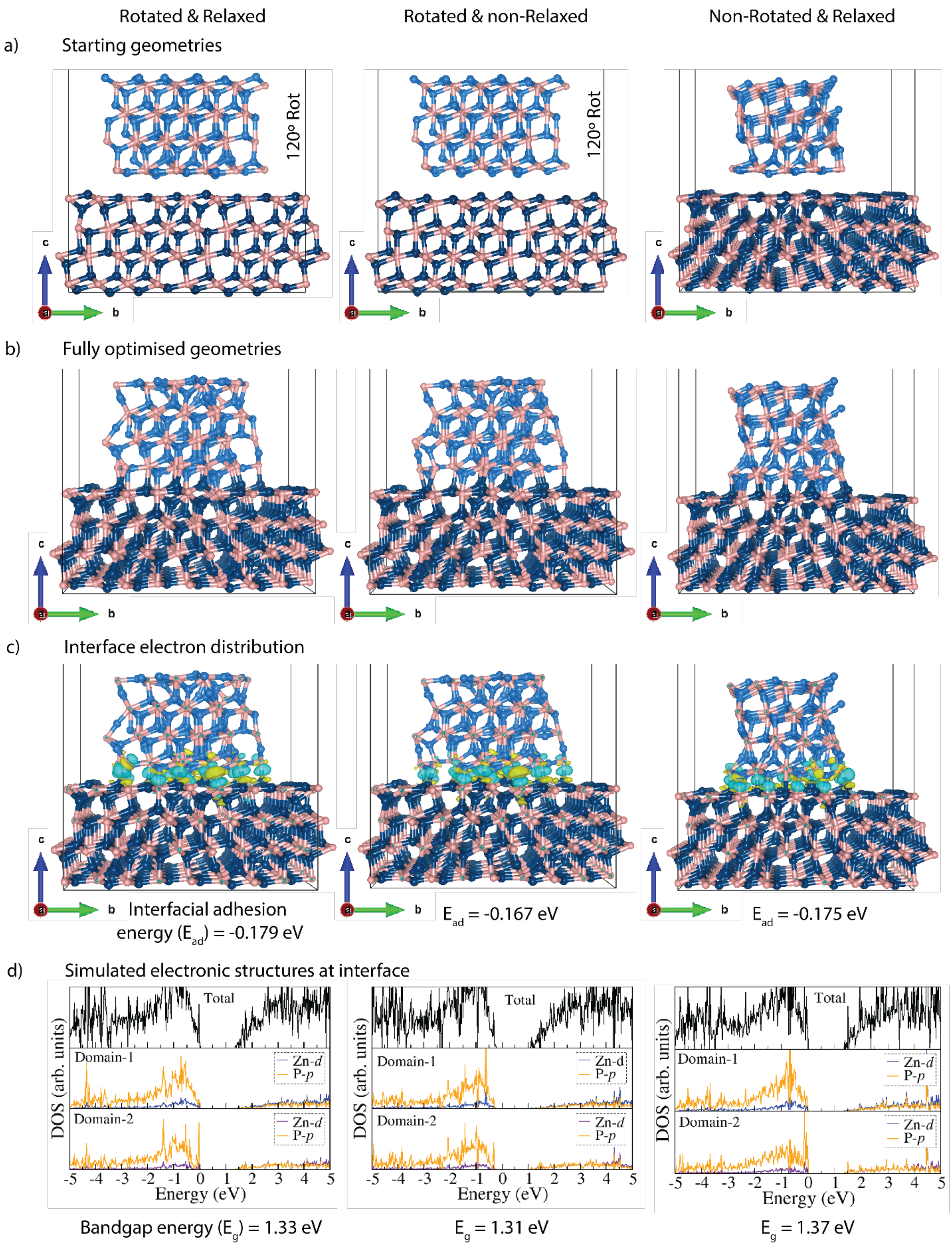

Figure S8. Additional DFT simulations of various (un)rotated interface configurations.

In Figure S9 we report a Zn3P2 heterotwin superlattice nanowire highlighting the presence of rotated domains.

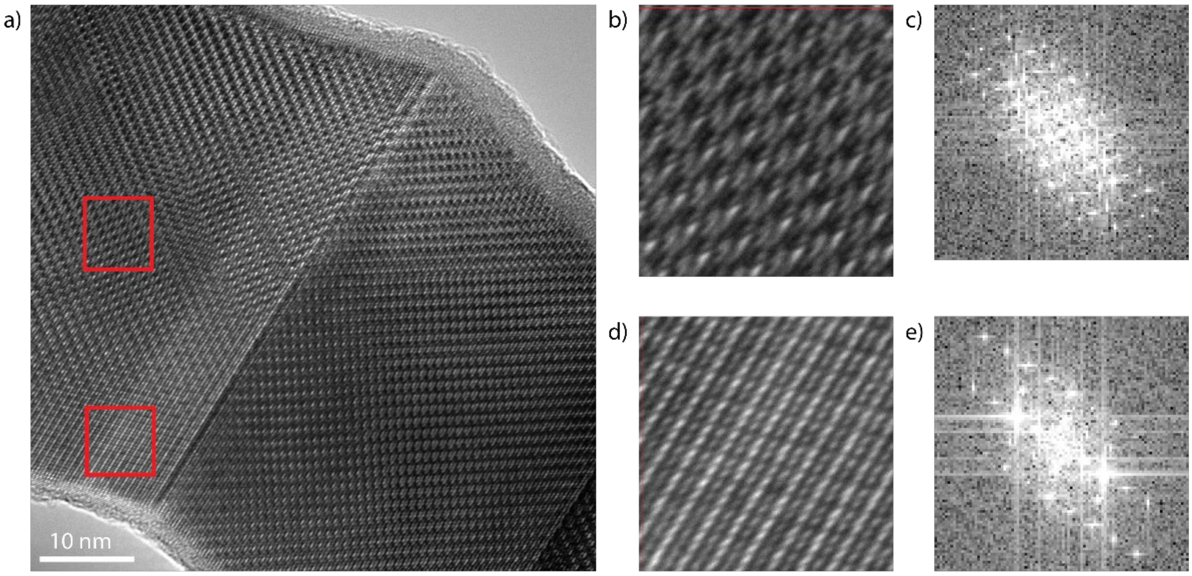

Figure S9. (a) HRTEM image of a Zn_3P_2 heterotwin superlattice nanowire, with boxes highlighting the areas shown in b and d. (b) HRTEM pattern of a <100>$_{Zn3P2}$ zone axis and (c) corresponding power spectrum. (d) HRTEM pattern of a <111>$_{Zn3P2}$ zone axis and (d) corresponding power spectrum. FFT filtered mapping is not presented due to it not being as clear due to the superimposed power spectra of the neighbouring rotated (twinned) sections.
